# First person – Sarah Haßdenteufel

**DOI:** 10.1242/bio.042549

**Published:** 2019-03-15

## Abstract

First Person is a series of interviews with the first authors of a selection of papers published in Biology Open, helping early-career researchers promote themselves alongside their papers. Sarah Haßdenteufel is first author on ‘ The signal peptide plus a cluster of positive charges in prion protein dictate chaperone-mediated Sec61 channel gating’, published in BiO. Sarah conducted the research described in this article while a postdoc in Prof. Dr Richard Zimmermann's lab at Saarland University, Germany. She is now a postdoc in the lab of Dr Sven Lang at Saarland University, investigating protein transport into the human endoplasmic reticulum.


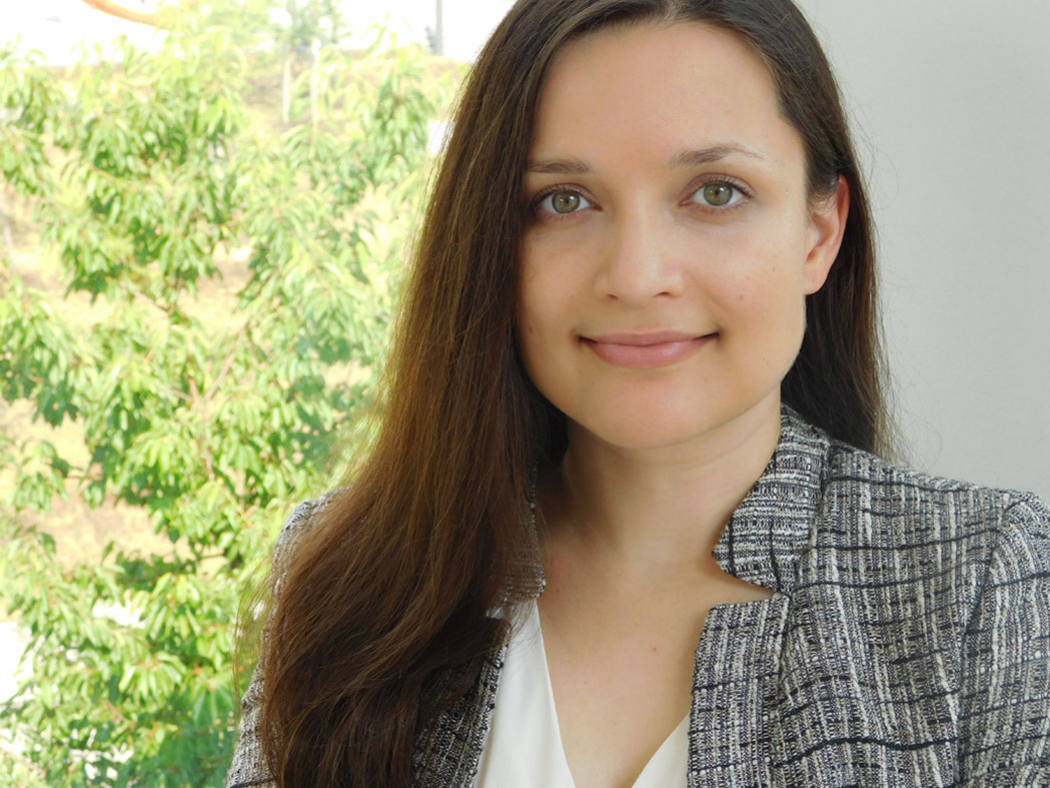


**Sarah Haßdenteufel**

**What is your scientific background and the general focus of your lab?**

I did my diploma in Biology at the Saarland University in Germany. After some training in epigenetics and structural biology, I found myself in medical biochemistry, where I graduated at the end of 2017 in the lab of Prof. Dr Richard Zimmermann. This paper is the first paper I wrote as postdoc in his lab. It's a first and extremely exciting for me. Our focus: how is the central Sec61 protein conducting channel in the human ER membrane regulated? To answer this question, we track traffic in both directions; passage of newly synthesized proteins from the cytosol into the ER lumen and efflux of calcium ions from the ER storage into the cytosol. The presented work is part of our recent findings that Sec61 requires regulation by a chaperone-pair when highly charged proteins are translocated.

**How would you explain the main findings of your paper to non-scientific family and friends?**

We look into the way some proteins must look because of their function and challenges in the process of their building at a step where it has not been expected. As an analogy; think of a cell as a city with V.I.P clubs. A protein that is heavily charged or otherwise far from ideal has difficulty entering the compartment of the endoplasmic reticulum (ER), much like the dress code required for gaining entrance to a club on a Saturday night (see the lady with static hair depicted in the comic!). We identified a gate-keeper that helps to open the club doors: the channel in the ER membrane.

**What are the potential implications of these results for your field of research?**

They change the view on why those gate-keepers exist – why the translocation machinery is so complex. One model beautifully stated the potential for regulation when proteins with an aberrant address tag rely on auxiliary translocation components. Our results now point to an additional scenario based on yet unknown difficulties in the translocation process. From a broader perspective, we started asking what else other than the address tag impacts translocation through the channel – and we found that those gate-keepers compensate for deleterious features in the mature protein. Considering the medical aspect of our model protein, we give one explanation for putative failure in ER import of the prion protein which is associated with neurodegeneration. Furthermore, we provide a novel example of a non-canonical chaperone-function in assisting of Sec61 channel opening.

**What has surprised you the most while conducting your research?**

The paradox of energy. I noticed that quite often you feel tired not because your work-load is too high, but because you have lost yourself in work which makes you stay at home after lab time, being too tired for hobbies and friends. For me, the best thing is to do exactly the opposite. So, go out, start painting, run, play! Exploring in and out of the lab keeps us healthy and guarantees best the success of our research. It sounds exhausting but gives you energy for coming back to work. I call it a paradox because you gain something by spending it. Maybe that's what the cell does! It spends energy keeping things under control. Having our presented model in mind, the cell spends energy in order to reduce the energetic barrier for channel opening.

“Exploring in and out of the lab keeps us healthy and guarantees best the success of our research.”

**What changes do you think could improve the professional lives of early-career scientists?**

That is a big subject. I see young group leaders juggling with a range of tasks, bureaucracy and limitations (money, for one), whilst they are still caring for their students ­– keeping at the aim of being a good mentor. Let's be proud together and love what we do. Only then may others become aware of the value and impact of our work so that the conditions for scientists can improve.

**Figure:**
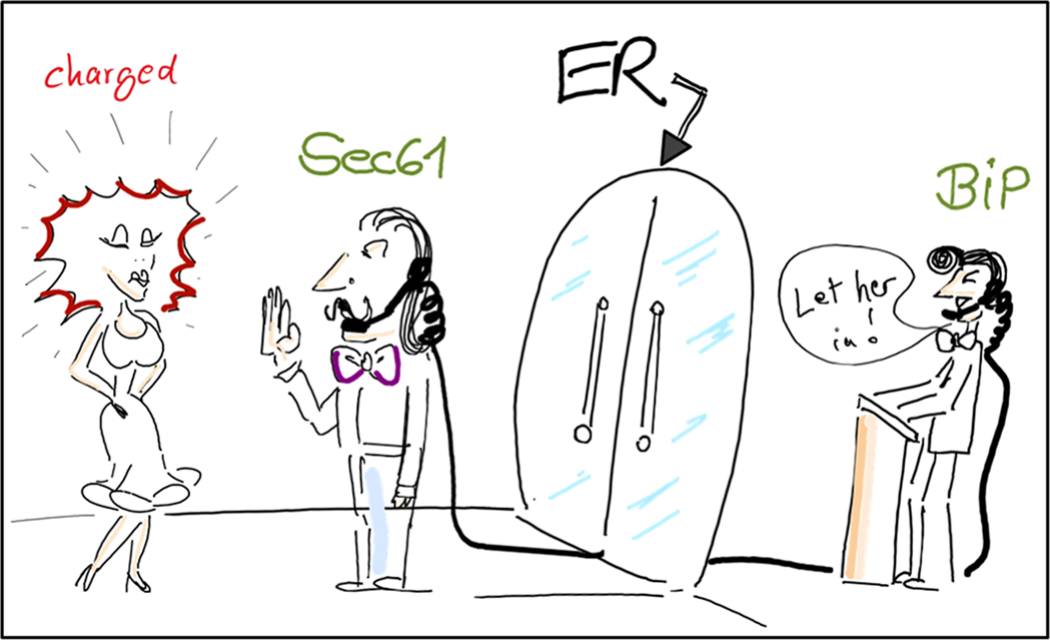
**Cellular organelle in analogy to a V.I.P club.** A protein that is heavily charged or otherwise far from ideal (lady in red) has difficulty entering the compartment of the endoplasmic reticulum (ER), much like with a dress code required for gaining entrance to a club on a Saturday night. The only way ‘in’ is via the help of the gate-keeping protein BiP, an ER lumenal Hsp70 chaperone who mediates the opening of the Sec61 protein-conducting channel in the ER membrane.

“Let's be proud together and love what we do.”

**What's next for you?**

After the current excursion into x-ray crystallography for studying protein–protein interaction, I will go to Israel for a second postdoc. During my PhD I learned what it means to biochemically dissect a certain phenomenon in human cells and I loved digging into the details. Now, I would like to learn the opposite approach – zooming out for the big picture and tackling an important question with genetic high throughput screens in yeast. From the previous manual screening ­– which was based on immense pipetting work – it will help me to deal with immense data sets from automated microscopy-based screens. The diversity of questions that can be addressed is fascinating – just by changing set-up and study design. The output of the assay is the same. I also find those screens attractive because they enable us to open new areas within the field which may provide a basis for mechanistic follow-up studies. But besides that, I like that the PI is very dedicated in training all aspects of becoming an independent scientist.

**What drives you in your personal life and in your professional career?**

Understanding things makes us happy, biochemically because of the release of dopamine, and mentally because it gives us a sense of security and control. In addition, I like travelling and seeing the world! I am very enthusiastic and tend to be impressed by many things – data, thoughts, (free-spirited, funny) people, the new. Nothing is as it seems. Nothing has to be the way you are used to.

**What has impressed you the most while conducting your research?**

I was grateful to meet diverse people from around the world in my lab, collaborations and on conferences. The dedication of scientists who literally say they work for making the world and our lives better. The collegial spirit of scientists who support each other across research groups and research fields. Lastly, the young group leaders I mentioned above.
